# DOPA-decarboxylase is elevated in CSF, but not plasma, in prodromal and de novo Parkinson’s disease

**DOI:** 10.1186/s40035-024-00421-0

**Published:** 2024-06-11

**Authors:** Ellen Appleton, Shervin Khosousi, Michael Ta, Michael Nalls, Andrew B. Singleton, Andrea Sturchio, Ioanna Markaki, Wojciech Paslawski, Hirotaka Iwaki, Per Svenningsson

**Affiliations:** 1 Laboratory of Translational Neuropharmacology, Department of Clinical Neuroscience, Karolinska Institutet, Stockholm, Sweden; 2 Center for Alzheimer’s and Related Dementias, National Institute On Aging and National Institute of Neurological Disorders and Stroke, National Institutes of Health, Bethesda, MD USA; 3 DataTecnica LLC, Washington, DC, USA; 4 Laboratory of Neurogenetics, National Institute On Aging, National Institutes of Health, Bethesda, MD USA; 5 James J. and Joan A. Gardner Family Center for Parkinson’s Disease and Movement Disorders, Department of Neurology, University of Cincinnati, Cincinnati, OH USA; 6 Basic and Clinical Neuroscience, King’s College London, London, UK

Parkinson’s disease (PD) diagnosis is based solely on clinical presentation [1]. Therefore, diagnostic and prognostic biomarkers are needed. In recent years, seeding aggregation assays (SAA) have demonstrated the ability to discriminate PD from controls, showing also potential as a predictive marker [2]. Furthermore, the development of modern proteomic techniques such as the proximity extension assay (PEA) has enabled high throughput, highly sensitive multiplexing studies to become more prevalent. In the past year, several such studies identified DOPA decarboxylase (DDC) as an analyte of particular interest, demonstrating consistent elevation in Lewy body diseases and promising diagnostic potential. Increased DDC has been found in the cerebrospinal fluid (CSF) [3–5] and plasma [4] of both PD [3–5] and dementia with Lewy bodies [4, 5] as well as atypical PD [3, 4], but not in non-parkinsonian neurodegenerative conditions such as Alzheimer’s disease [5]. Intriguingly, CSF DDC is elevated in SAA-positive subjects, independent of Lewy body disease diagnosis [4].

This study aimed to evaluate CSF and plasma levels of DDC in prodromal, de novo, and treated PD patients and controls using PEA in three independent cohorts, the Parkinson’s Progression Markers Initiative (PPMI) (de novo PD *n* = 74 CSF, *n* = 78 plasma; prodromal PD *n* = 51 CSF, *n* = 62 plasma; and control *n* = 130 CSF, *n* = 130 plasma); Parkinson’s Disease Biomarkers Program (PDBP) (PD *n* = 84 CSF, *n* = 84 plasma; and control *n* = 54 CSF, *n* = 54 plasma); and Biopark (PD *n* = 120 [27 de novo] CSF, *n* = 238 [26 de novo] plasma; and control *n* = 69 CSF, *n* = 50 plasma) (Additional file 1: Table S1 and Supplementary Methods).

Differential abundance analysis of 1463 proteins in the CSF revealed DDC to be the most significantly changed protein in PD (PDBP), prodromal PD and de novo PD (PPMI) compared to controls. Focused investigation of PD CSF samples from Biopark using the Olink “Metabolism” panel (91 proteins) similarly identified DDC as the most increased analyte (Fig. 1a–g). Further analysis revealed similar elevations of CSF DDC in both prodromal and de novo PD in the PPMI cohort (Fig. 1f).

**Fig. 1:**
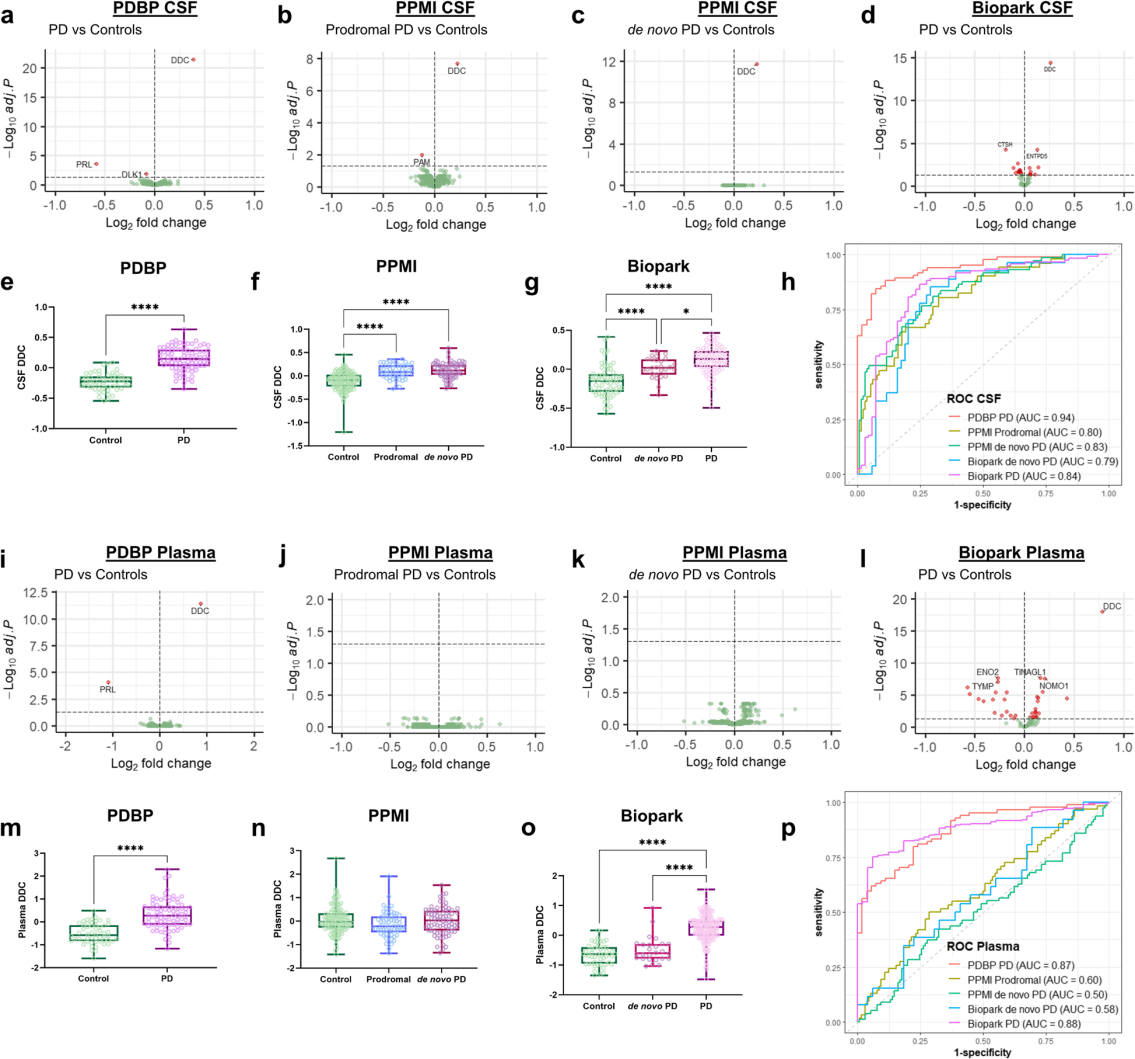
CSF and plasma DDC levels in prodromal, de novo and treated PD. **a**–**d** Volcano plots showing differential abundance of 1463 proteins in the CSF, corrected for age and sex. Axes display Log_2_ fold change and Benjamini–Hochberg adjusted *P*-values. Plots show PD (*n* = 84) versus controls (*n* = 54) in the PDBP cohort (**a**), prodromal PD (*n* = 51) versus controls (*n* = 130) (**b**) and de novo PD (*n* = 74) versus controls (*n* = 130) (**c**) in the PPMI cohort, and PD (*n* = 120) versus controls (*n* = 69) (66 proteins) in the Biopark cohort (**d**). **e**–**g** Box-plots of age- and sex-adjusted CSF DDC levels in PD and controls from the PDBP cohort (independent *t*-test) (**e**), prodromal, de novo PD and control samples from the PPMI cohort (ANOVA, Tukey post-hoc test) (**f**), and de novo PD, PD and control samples from the Biopark cohort (ANOVA, Tukey post-hoc test) (**g**). **h** Receiver operating characteristic (ROC) curves for CSF DDC levels with area under the curve (AUC) for prodromal or PD vs controls in each cohort. **i**–**l** Volcano plots showing differential abundance of 1463 proteins in plasma, corrected for age and sex. Axes display Log_2_ fold change and Benjamini–Hochberg adjusted *P*-values. Plots show PD (*n* = 84) versus controls (*n* = 54) in the PDBP cohort (**i**), prodromal PD (*n* = 62) versus controls (*n* = 130) (**j**) and de novo PD (*n* = 78) versus controls (*n* = 130) (**k**) in the PPMI cohort, and PD (*n* = 238) versus controls (*n* = 50) (71 proteins) in the Biopark cohort (**l**). **m**–**o** Box-plots of age- and sex-adjusted plasma DDC levels in PD and controls from the PDBP cohort (independent *t*-test) (**m**), prodromal, de novo PD and control samples from the PPMI cohort (ANOVA, Tukey post-hoc test) (**n**), and de novo PD, PD and control samples from the Biopark cohort (ANOVA, Tukey post-hoc test) (**o**). **p** ROC curves for plasma DDC levels with AUC for prodromal or PD vs controls in each cohort. *P < 0.05, ****P < 0.0001

Receiver operating characteristic (ROC) curves were constructed to assess the discriminatory capacity of CSF DDC (Fig. 1h, Table S2). DDC was able to discriminate PD from controls with area under the curve (AUC) of 0.79–0.94 in three cohorts. The highest AUC was in the PDBP cohort where most PD patients were on treatment, and the lowest in the de novo, unmedicated PD patients from PPMI and Biopark. Prodromal PD showed comparable results, with an AUC of 0.80.

As most controls and de novo PD patients in the PPMI cohort had data on SAA status available, we analysed its association with DDC levels in CSF. SAA positivity was associated with higher DDC levels in both PD and the few SAA^+^ controls. Moreover, the few SAA^-^ PD subjects had below-average levels of DDC in the PD group (Fig. S1).

The Olink analysis was repeated in plasma samples from the same three cohorts (Fig. 1i–o). As in CSF, DDC was the most significantly increased protein in PD plasma samples from the PDBP and Biopark cohorts, compared to controls. However, DDC was not altered in prodromal or de novo PD from the PPMI cohort (Fig. 1n). Additionally, separation of the Biopark PD samples into de novo and treated PD also demonstrated no significant plasma DDC increase in de novo PD vs controls (Fig. 1o).

Furthermore, ROC analysis showed plasma DDC levels to be capable of differentiating between PD and controls in both the PDBP and the Biopark (AUC = 0.87–0.88) cohorts. This was not replicated in de novo PD from PPMI and Biopark (AUC = 0.50–0.58), or prodromal PD (AUC = 0.60) (Fig. 1p, Table S2).

The three cohorts are heterogeneous in terms of PD stage and medication and further analyses revealed that disease duration and levodopa equivalent daily dose in the PDBP cohort were positively correlated with both CSF and plasma DDC levels (Fig. S2). The PPMI de novo PD cohort was followed over a period of four years, during which time many participants began PD treatment (Fig. S3). From this longitudinal dataset, we selected the latest time point for each patient (average disease duration 2.8 years for CSF, 2 years for plasma), in order to further explore the influence of medication on DDC levels. As in the Biopark cohort, plasma DDC was significantly increased in treated PD samples only, whilst CSF DDC was increased in both treated and untreated PD (Fig. S3). The increase in plasma DDC appeared to be driven by levodopa/DDC-inhibitor treatment specifically, as the same effect was not observed in those receiving only dopamine agonists, an effect mirrored in the PDBP cohort (Figs. S3 and S4).

After correcting for age and sex, a weak correlation between CSF DDC and Unified Parkinson’s Disease Rating Scale 3 (UPDRS-3) score (*R* = 0.23, *P* = 0.048) was found in de novo PD patients from the PPMI cohort, but it was not reproduced in other cohorts (Fig. S5). No other correlations were identified in CSF and/or plasma.

By utilising PEA analysis in three independent cohorts, we have provided further evidence of the efficacy of DDC as a diagnostic PD biomarker. Of particular importance, assessment of prodromal and de novo PD patients from the PPMI and Biopark cohorts demonstrated that CSF DDC has diagnostic capacity in early, untreated PD, and even before PD diagnosis.

The Biopark, PPMI, and PDBP cohorts are heterogeneous with regard to disease stage, disease duration, and PD treatment status, features which were shown to correlate with DDC levels. Levodopa had a small influence on CSF DDC. For example, the highest AUC was in the PDBP cohort where most PD patients were on treatment, and the lowest were in the unmedicated, de novo PD patients from the PPMI and Biopark cohorts. Nevertheless, DDC is an excellent CSF biomarker across cohorts, independent of treatment.

We speculate that the increase of DDC in the CSF could be a compensatory mechanism as suggested in animal models [6]. Another possible reason could be the release of DDC from dying monoaminergic neurons. DDC has limited correlation with clinical features; in fact, only a mild correlation was found between DDC and UPDRS-3 total score, questioning its validity as a robust prognostic biomarker. Moreover, it has been shown that different DDC polymorphisms can affect the levodopa response, adding another layer of complexity for the clinical correlation [7].

Whilst plasma DDC was very effective in discriminating between treated PD and controls, this did not extend to prodromal and de novo PD. As has been shown previously, plasma DDC levels are affected by levodopa/DDC-inhibitor treatment [8]. Intriguingly, this effect was not seen in subjects treated with dopamine agonists. It would be interesting to investigate what other factors related to PD medication affect DDC levels, including the type and dose of DDC inhibitors, the effect of MAO/COMT inhibitors, and different dopamine agonists.

Our paper adds novelty to the recent findings [3–5] in which higher DDC levels have been found in subjects with Lewy body dementia and atypical parkinsonism, corroborating the importance of DDC in the diagnosis of parkinsonian syndromes.

A strength of this study is the large sample size and validation of the results using several cohorts. However, this could be strengthened further using orthogonal methods to corroborate DDC measurements.

In conclusion, our data show that CSF DDC has strong biomarker potential in PD. Since we found alterations even in the prodromal phase, DDC could be also an excellent biomarker for the identification of patients suitable for clinical trials.

### Supplementary Information

**Additional file 1**: **Figure S1**. The relationship between SAA and DDC in the PPMI cohort. **Figure S2**. Spearman’s rank correlation between CSF and plasma DDC levels with disease duration and LEDD in the PDBP cohort. **Figure S3**. PPMI cohort - last time point sampled. **Figure S4**. PD Treatment in the PDBP cohort. **Figure S5**. Spearman’s rank correlation between CSF and plasma DDC levels with UPDRS 3 score in PD patients from each cohort. **Table S1**. Cohort distributions. **Table S2**. ROC analysis. Methods.

## Data Availability

Data used in the preparation of this article were obtained [on October 31, 2022.] from the Parkinson’s Progression Markers Initiative (PPMI) database (www.ppmi-info.org/access-data-specimens/download-data), RRID:SCR_006431. The PDBP data were obtained from the DMR (data management resources (https://pdbp.ninds.nih.gov/)) on May 27th 2021. The Biopark dataset analysed during the current study is available from the corresponding author on reasonable request.

## Abbreviations

PD: Parkinson’s disease
SAA: Seeding aggregation assay
PEA: Proximity extension assay
DDC: DOPA decarboxylase
CSF: Cerebrospinal fluid
PPMI: Parkinson’s Progression Markers Initiative
PDBP: Parkinson’s Disease Biomarkers Program
ROC: Receiver operating characteristic
AUC: Area under the curve
UPDRS: Unified Parkinson’s disease rating scale
