# Supplementary material for: DOPA-decarboxylase is elevated in CSF, but not plasma, in prodromal and de novo Parkinson’s disease
Source: Transl Neurodegener. 2024 Jun 11;13:31. doi: 10.1186/s40035-024-00421-0 (PMC11165760; doi:10.1186/s40035-024-00421-0)
Supplement: Supplementary file 1 — Additional file 1: Figure S1. The relationship between SAA and DDC in the PPMI cohort. Figure S2. Spearman’s rank correlation between CSF and plasma DDC levels with disease duration and LEDD in the PDBP cohort. Figure S3. PPMI cohort - last time point sampled. Figure S4. PD Treatment in the PDBP cohort. Figure S5. Spearman’s rank correlation between CSF and plasma DDC levels with UPDRS 3 score in PD patients from each cohort. Table S1. Cohort distributions. Table S2. ROC analysis. Methods. [file 40035_2024_421_MOESM1_ESM.docx]

**SUPPLEMENTARY MATERIALS**

**
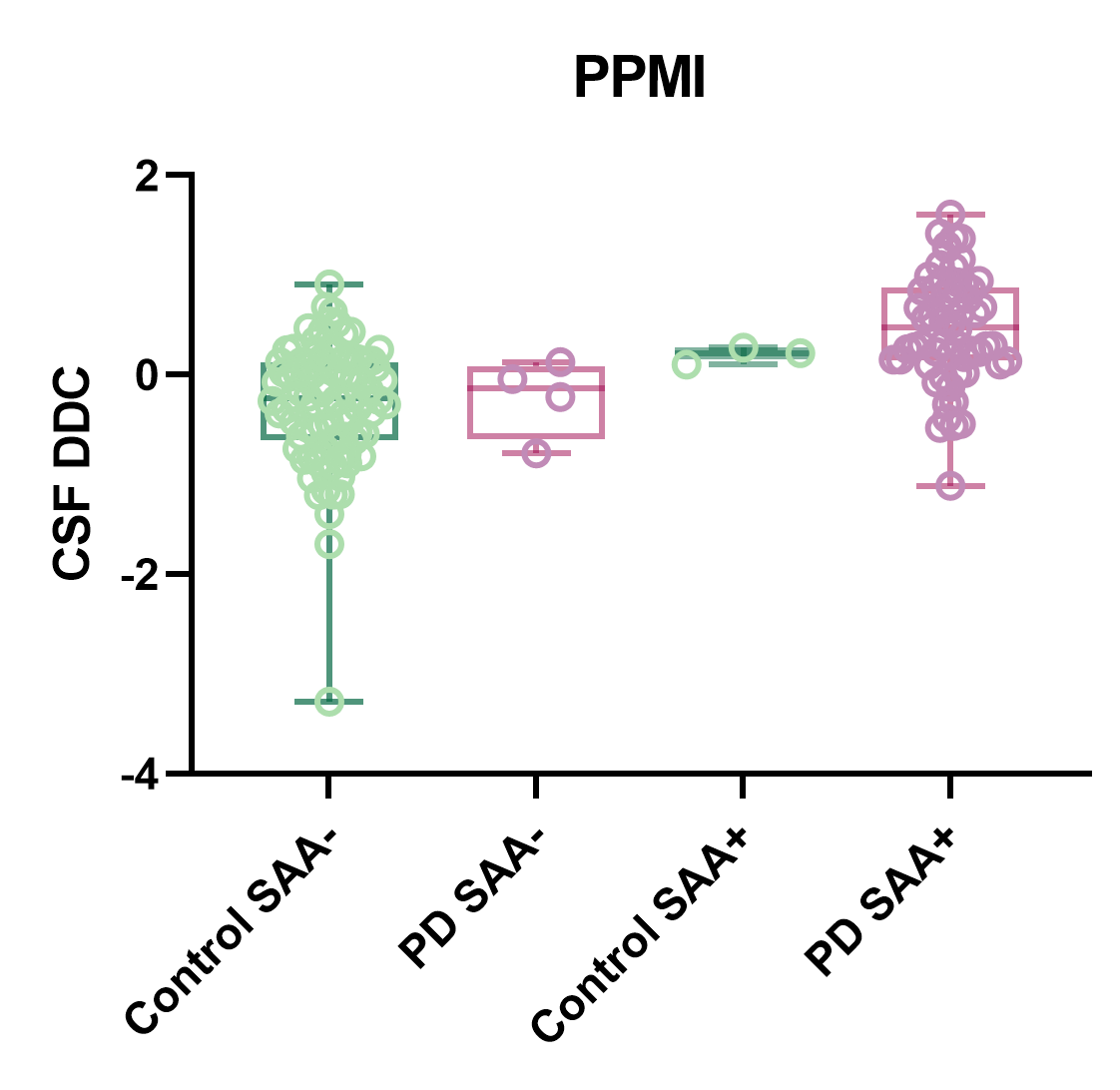
**

**Figure S1: The relationship between SAA and DDC in the PPMI cohort.** Box-plots of age and sex adjusted CSF DDC levels in PD and controls with positive or negative seeding aggregation assay (SAA) status.


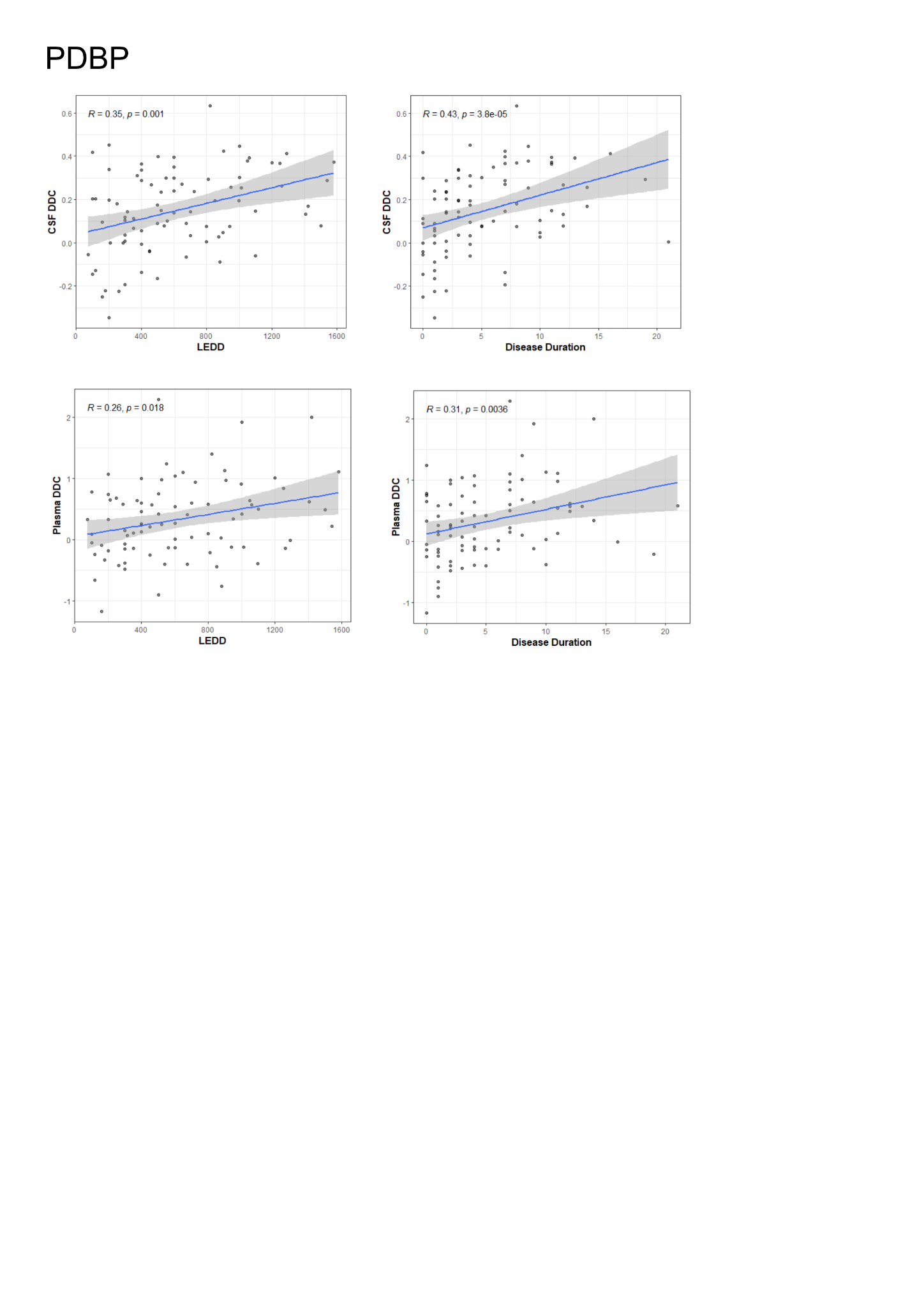


**Figure S2:** Spearman’s rank correlation between CSF and plasma DDC levels with disease duration and LEDD (levodopa equivalent daily dose) in the PDBP cohort.


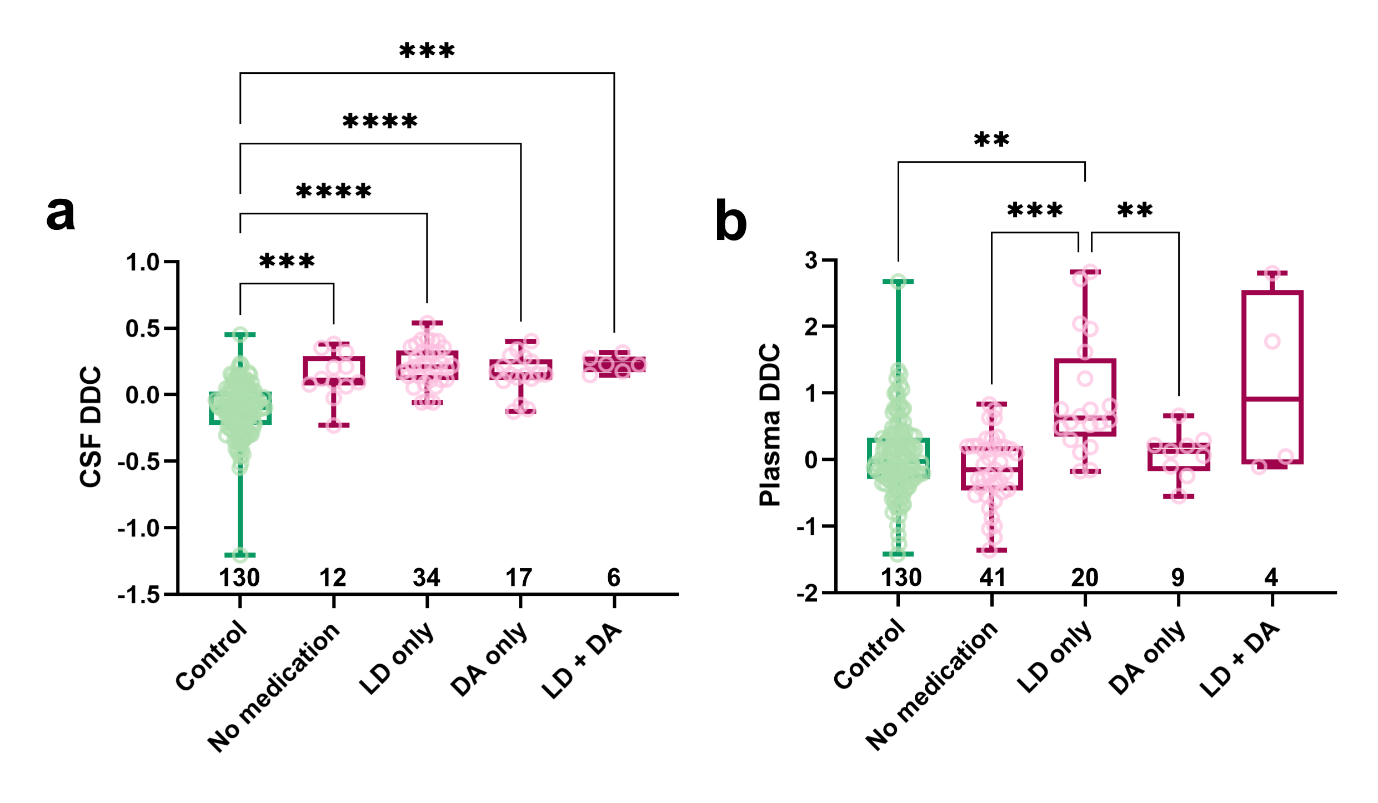


**Figure S3: PPMI cohort - last time point sampled.** Comparison of age- and sex-adjusted DDC levels in CSF (**a**) and plasma (**b**) across controls, untreated and treated PD. Treated PD samples were divided into those receiving levodopa (LD), dopamine agonists (DA) or both (LD+DA). The number of patients in each treatment group is indicated above the x-axis. A small number of patients were excluded from this analysis due to incomplete treatment information (5/74 in CSF and 4/78 in plasma). Significant alterations in CSF DDC were determined using one-way ANOVA and Tukey’s multiple comparison test (adjusted *P*-value). Significant alterations in plasma DDC were determined using Brown-Forsythe one-way ANOVA and Dunnett’s T3 multiple comparison test, due to violation of the assumption of homogeneity of variance. Significant differences are indicated on the graph (***P* <0.01, ****P* <0.001, *****P* <0.0001). All other comparisons were non-significant.


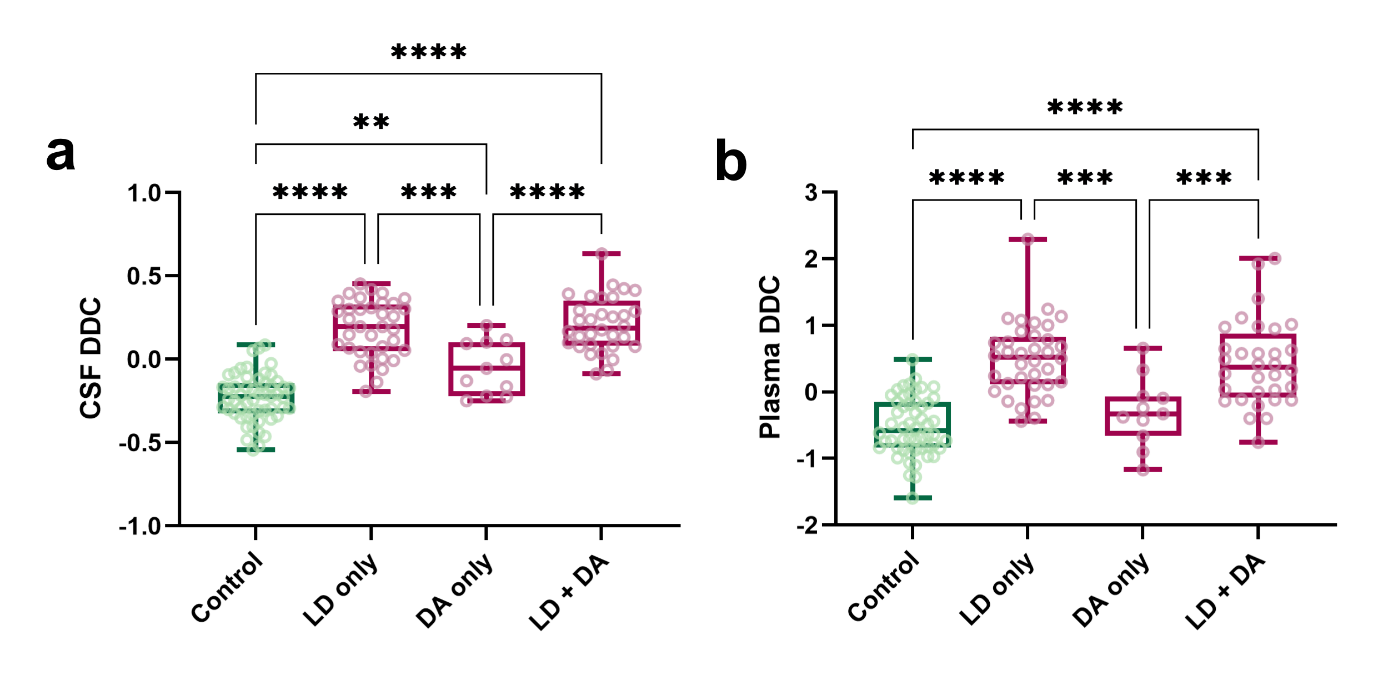


**Figure S4: PD Treatment in the PDBP cohort.** Comparison of age and sex adjusted DDC levels across controls and treated PD using one-way ANOVA and Tukey’s multiple comparison test (adjusted p-value) in CSF (**a**) and plasma (**b**). Treated PD samples are divided into those receiving levodopa (LD), dopamine agonists (DA) or both (LD+DA). Significant differences are indicated on the graph (***P* <0.01, ****P* <0.001, *****P* <0.0001). All other comparisons were non-significant.

*
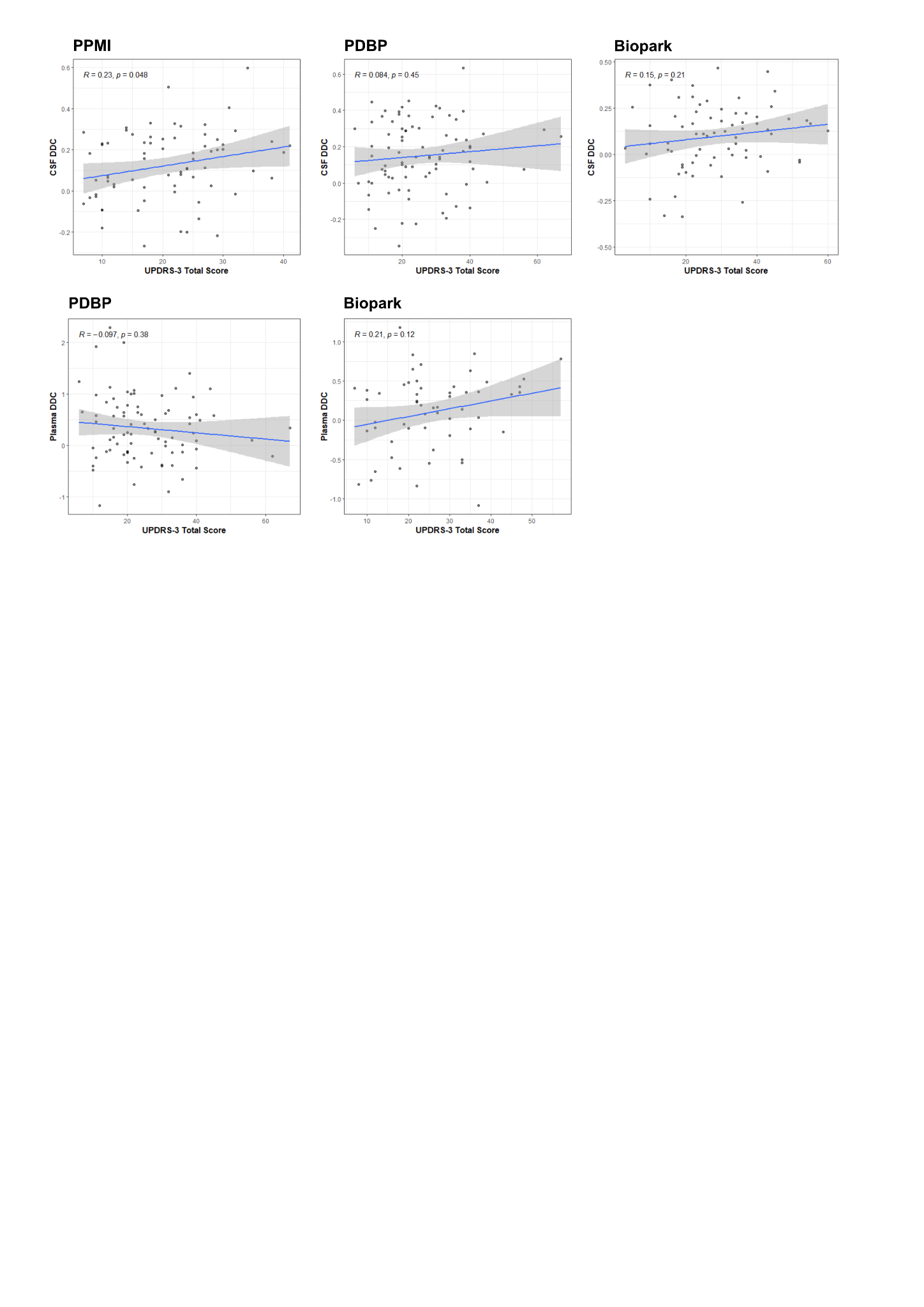
*

**Figure S5:** Spearman’s rank correlation between CSF and plasma DDC levels with UPDRS 3 score in PD patients from each cohort.

**Table S1: Cohort distributions.**

| **Cohort** | **Disease group** | **Biofluid** | ***N*** | **Sex (%M)** | **Age (Years)** | **Disease Duration (Years)** | **UPDRS-3 Total** |
| --- | --- | --- | --- | --- | --- | --- | --- |
| Biopark | Control | CSF | 69 | 48 | 64.7(9.9) |  |  |
|  |  | Plasma | 50 | 57 | 64(8) |  |  |
|  | PD | CSF | 120 (27)^dn^ | 66 (63)^dn^ | 65.4(10.6) (63.2(12.7))^dn^ | 2.6(3.8) (0.5(0.45))^dn^ | 28.5(12.8) (23.8(13.7))^dn^ |
|  |  | Plasma | 238 (26)^dn^ | 63 (63)^dn^ | 66.3(10) (66(11.1))^dn^ | 4.3(4.6) (0.7(0.7))^dn^ | 25.5(11.4) (18.9(6.8))^dn^ |
| PPMI | Control | CSF | 130 | 65 | 61.2(10.3) |  |  |
|  |  | Plasma | 130 | 65 | 61.2(10.3) |  |  |
|  | Prodromal | CSF | 51 | 75 | 68.5(5.2) |  |  |
|  |  | Plasma | 62 | 77 | 69.5(5.7) |  |  |
|  | *de novo* PD | CSF | 74 | 69 | 61.7(10.4) | 0.05(0.17) | 20.2(8.7) |
|  |  | Plasma | 78 | 69 | 61.5(10.7) | 0.04(0.16) | 20.9(8.6) |
| PDBP | Control | CSF | 54 | 52 | 64.6(10.9) |  |  |
|  |  | Plasma | 54 | 52 | 64.6(10.9) |  |  |
|  | PD | CSF | 84 | 68 | 64.4(8) | 5.3(4.6) | 25.5(12) |
|  |  | Plasma | 84 | 68 | 64.4(8) | 5.3(4.6) | 25.5(12) |

Information regarding each cohort used in this study, described in detail in Methods. *N* = total number of samples. Age, disease duration and Unified Parkinson’s Disease Rating Scale 3 (UPDRS-3) values are shown as mean and standard deviation (in brackets). Sex is shown as percentage male. Biopark PD patients had a range of disease durations and treatments. In Figure 1(g),(o) *de novo* PD patients (a subset of Biopark PD) were analysed separately. The distributions of this subset are also shown here, indicated by the superscript dn (de novo).

**Table S2. ROC analysis.**

| **ROC** | **AUC** | **Sensitivity** | **Specificity** | **PPV** | **NPV** | **Threshold** | **CV AUC** | **CV AUC SE** | **CV AUC CI** |
| --- | --- | --- | --- | --- | --- | --- | --- | --- | --- |
| PDBP PD vs Control CSF | 0.94 | 0.85 | 0.93 | 0.95 | 0.79 | -0.05 | 0.94 | 0.02 | 0.90-0.98 |
| PPMI Prodromal vs Control CSF | 0.8 | 0.8 | 0.68 | 0.49 | 0.9 | -0.03 | 0.81 | 0.04 | 0.74-0.88 |
| PPMI de novo PD vs Control CSF | 0.83 | 0.77 | 0.75 | 0.63 | 0.85 | 0.02 | 0.85 | 0.03 | 0.75-0.88 |
| Biopark de novo PD vs Control CSF | 0.79 | 0.85 | 0.71 | 0.53 | 0.92 | -0.1 | 0.8 | 0.04 | 0.71-0.89 |
| Biopark PD vs Control CSF | 0.84 | 0.87 | 0.75 | 0.86 | 0.76 | -0.07 | 0.82 | 0.03 | 0.76-0.89 |
| PDBP PD vs Control plasma | 0.87 | 0.8 | 0.78 | 0.85 | 0.71 | -0.14 | 0.88 | 0.03 | 0.82-0.94 |
| PPMI Prodromal vs Control plasma | 0.6 | 0.5 | 0.72 | 0.46 | 0.75 | -0.24 | 0.6 | 0.04 | 0.51-0.68 |
| PPMI de novo PD vs Control plasma | 0.5 | 0.42 | 0.68 | 0.44 | 0.66 | 0.2 | 0.43 | 0.04 | 0.35-0.51 |
| Biopark de novo PD vs Control plasma | 0.58 | 0.88 | 0.31 | 0.4 | 0.83 | -0.87 | 0.55 | 0.06 | 0.42-0.67 |
| Biopark PD vs Control plasma | 0.88 | 0.75 | 0.94 | 0.98 | 0.44 | -0.16 | 0.87 | 0.02 | 0.83-0.91 |

Fit statistics for each ROC analysis performed, including area under the curve (AUC), sensitivity, specificity, positive predictive value (PPV), negative predictive value (NPV) and the associated threshold, defined using Youden’s J statistic. AUC was further evaluated using 10-fold cross validation (CV), with the mean, standard error (SE) and confidence interval (CI) displayed.

**METHODS**

Cohorts

The samples and data utilised in this study originated from three independent cohorts, namely Biopark, PPMI and PDBP.

Biopark

Biopark is a Swedish Parkinson focused cohort managed under Region Stockholm, continuously recruiting individuals with Parkinson’s disease, atypical parkinsonisms, and healthy controls since 2011. Patients are clinically assessed and leave blood samples at regular intervals. Additionally, CSF was collected from PD patients at some time point. PD patients were diagnosed by a movement disorder specialist and met the UK Brain Bank criteria. Control subjects were individuals free of any neurological disease, and selected if their age and sex were close to those in the PD group. Sample collection for CSF [1] and blood [2], and clinical assessments in the Biopark cohort were carried out as previously described [3].

PPMI

The Parkinson’s Progression Markers Initiative (PPMI) is an observational clinical study that facilitates the identification of prognostic and diagnostic PD biomarkers through a large database of clinical assessments, imaging studies and biological specimens from 33 international clinical sites. For up-to-date information on the study, including clinical assessment and biofluid collection protocols, visit [www.ppmi-info.org](http://www.ppmi-info.org).

For this study, we had access to proteomics analysis of CSF and plasma samples from control, prodromal and *de novo* PD participants (Figure 1, Table S1), as well as complementary patient information and clinical assessments (including UPDRS, MoCA). PD patients have a clinical diagnosis as well as a positive dopamine transporter (DAT) SPECT. Prodromal participants include people with rapid eye movement sleep behaviour disorder (RBD), hyposmia, deficits on DaTSCAN and/or with genetic risk variants. Control subjects did not have any neurological disease nor any first-degree relatives with PD. PPMI is a longitudinal study and we also had access to follow-up clinical and proteomic assessments up to 4 years following diagnosis for the PD participants, at which point some participants had begun to receive PD treatments (Figure S3).

The α-synuclein seed amplification assay (SAA) results of PPMI CSF samples were available [4], and shown in Figure S1. SAA can be used to detect misfolded α-synuclein, a central feature of PD and other synucleinopathies. In brief, CSF samples are incubated with recombinant α-synuclein monomers which provide a substrate for endogenous α-synuclein aggregates present in CSF. These are then amplified by a cyclic process of fibril elongation and fragmentation. The aggregates can then be detected by conventional fluorescence using Thioflavin T. A time and signal threshold is set for when the sample is considered SAA positive, and SAA positivity suggests there exists synucleinopathy specific α-synuclein aggregates in the sample. Details of the Amprion α-synuclein SAA can be found in the original Nature Protocols paper from Concha-Marambio et al [5].

PDBP

The National Institute of Neurological Disorders and Stroke (NINDS) Parkinson’s Disease Biomarkers Program (PDBP) is a clinical study which aims to improve understanding of the pathophysiology of PD and its progression through a large cohort of PD patients and controls, from multiple sites. For up-to-date information on the study, including clinical assessment and biofluid collection protocols, visit <https://pdbp.ninds.nih.gov/>.

For this study, we had access to proteomics analysis of CSF and plasma samples from control and PD PDBP participants (Figure 1, Table S1), as well as complementary patient information and clinical assessments (including UPDRS, MoCA). PD patients were all clinically diagnosed, and controls were free from neurological conditions, and had no first-degree relatives with neurological conditions.

The data of PPMI and PDBP participants were obtained through AMP-PD programme’s data release version 3. The details of the collection and the harmonisation of data were described at the AMP-PD website (<https://www.amp-pd.org/>). For the drug usage data, we downloaded the data directly from each database repository. Data used in the preparation of this article were obtained [on October 31, 2022.] from the Parkinson’s Progression Markers Initiative (PPMI) database (www.ppmi-info.org/access-data-specimens/download-data), RRID:SCR_006431. This analysis used data openly available from PPMI (Tier 1). The PDBP data was obtained from the DMR (data management resources (<https://pdbp.ninds.nih.gov/>)) on May 27th 2021.

Data processing

In the PPMI and PDBP cohorts, Olink PEA was used to define the relative abundance (NPX values, https://olink.com/our-platform/our-pea-technology/data-generation-and-qc/) of 1463 proteins (Olink panels: Cardiometabolic, Neurology, Immunology and Oncology) in CSF and plasma samples. In the Biopark cohort, the relative abundance of 91 proteins (Olink metabolic panel) were analysed in CSF and plasma samples. Proteins with greater than 25% of values below the limit of detection were excluded from further analysis (66 proteins remaining in CSF and 71 in plasma).

The few flagged reads were removed from further analysis and samples were normalised to correct for technical variations. Where necessary, samples were normalised across Olink runs through the addition of per-protein normalisation constant, defined as the median difference between overlapping runs.

Differential expression analysis was conducted on the measured proteins for each cohort separately using multiple linear models, with diagnosis, age and sex as explanatory variables, and p-values were adjusted with the Benjamini-Hochberg method to control for multiple comparisons. All DDC values shown are corrected for age and sex.

**Supplementary references**

[1] Paslawski W, Khosousi S, Hertz E, Markaki I, Boxer A, Svenningsson P. Large-scale proximity extension assay reveals CSF midkine and DOPA decarboxylase as supportive diagnostic biomarkers for Parkinson's disease. Translational neurodegeneration. 2023;12(1):42.

[2] Khosousi S, Hye A, Velayudhan L, Bloth B, Tsitsi P, Markaki I, et al. Complement system changes in blood in Parkinson's disease and progressive Supranuclear Palsy/Corticobasal Syndrome. Parkinsonism & related disorders. 2023;108:105313.

[3] Markaki I, Ntetsika T, Sorjonen K, Svenningsson P. Euglycemia Indicates Favorable Motor Outcome in Parkinson's Disease. Movement disorders. 2021;36(6):1430-4.

[4] Siderowf A, Concha-Marambio L, Lafontant DE, Farris CM, Ma Y, Urenia PA, et al. Assessment of heterogeneity among participants in the Parkinson's Progression Markers Initiative cohort using α-synuclein seed amplification: a cross-sectional study. The Lancet Neurology. 2023;22(5):407-17.

[5] Concha-Marambio L, Pritzkow S, Shahnawaz M, Farris CM, Soto C. Seed amplification assay for the detection of pathologic alpha-synuclein aggregates in cerebrospinal fluid. Nature protocols. 2023;18(4):1179-96.
